# Patient handoffs among general surgery residents in Saudi Arabia: a cross-sectional study

**DOI:** 10.1186/s12909-022-03670-7

**Published:** 2022-08-18

**Authors:** Abdulaziz M. Saleem, Mai Kadi

**Affiliations:** 1 Department of General Surgery, Faculty of Medicine, King Abdulaziz University, Jeddah, Saudi Arabia; 2 Department of Community Medicine, Faculty of Medicine, King Abdulaziz University, Jeddah, Saudi Arabia

**Keywords:** Adverse events, Communication, Healthcare, Healthcare provider, Patient care, Patient handoff

## Abstract

**Background:**

Communication failure is a common cause of adverse events. An essential element of communication among health care providers is patient handoff. Patient handoff is defined as a practice whereby a health care provider transfers a patient’s care information to another health care provider to ensure the patient’s safety and continuity of care. To explore this practice, we assessed patient handoffs among general surgery residents in Saudi Arabia.

**Methods:**

A cross-sectional survey was conducted with individuals in accredited general surgery residency programs in Saudi Arabia between 2020 and 2021.

**Results:**

Participants comprised 118 general surgery residents: 66 (57.3%) were female; 67 (72.8%) did not receive any formal training on patient handoff; and 35 (38.8%) reported that they were sometimes interrupted during the patient handoff process. The most common reason for such interruptions was medical personnel paging. Furthermore, 60 (68.1%) general surgery residents stated that these interruptions led to a decreased quality of effective communication, 39 (44.3%) believed it led to decreased quality of patient care, 63 (71.5%) believed it led to the loss of some information related to patient handoff, and 16 (18.1%) believed it led to patient harm. Finally, 31 (34.4%) general surgery residents believed that the existing handoff system at their institutions neither adequately protected the patients’ safety nor allowed for continuity of care, and 51 (68%) reported that their institution did not have a standardized protocol for the verbal patient handoff process. There was a higher proportion of patients with minor harm among residents who did not, rarely or sometimes received verbal or written hand off instructions compare to those who did so always or most of the time (67% vs. 49%, respectively).

**Conclusion:**

The patient handoff process among general surgery residents in Saudi Arabia is subjective and is not standardized, and if not addressed, may lead to patient harm. Standardizing this process is paramount to improve patient safety.

**Supplementary Information:**

The online version contains supplementary material available at 10.1186/s12909-022-03670-7.

## Background

Patient handoff refers to a practice whereby a health care provider transfers a patient’s care information to another health care provider to ensure the patient’s safety and continuity of care. The safety and reliability of the U.S. health care system was determined to be at risk when, in 2000, the Institute of Medicine published a now-famous report showing that, on average, 98,000 deaths occur annually in U.S. hospitals as a direct result of medical errors [1]. A retrospective review that included 444 closed malpractice claims from four liability insurers found that communication breakdown was one of the system factors that led to medical errors [2]. A similar retrospective review of 51 hospitals in the state of New York showed that 26% of the systemic errors were due to inadequate reporting and communication [3]. A review of 7,926 patient medical records across 21 hospitals in the Netherlands found that the main cause of adverse events was knowledge-based mistakes and information-transfer problems [4]. In addition, medical errors can affect the overall health care system because of their cascading events: they ultimately increase the total cost and length of hospitalization as well as reduce trust in physicians, leading to job loss and decreased morale among physicians.

Failure to adequately communicate, known as a “communication breakdown,” has been repeatedly shown to be a leading cause of medical error in root cause analyses. Indeed, communication is critical to patient handoffs. When inadequately performed, patient handoffs can result in harm to patients. A cross-sectional study among surgical residents in the U.S. and Canada found that the existing patient handoff practices contributed to patient harm [5]. Another survey of general surgery residents and internal medicine residents at Massachusetts General Hospital showed that 59% of the residents reported that incomplete handoff had resulted in one or more patient harm events, of which 12% of the cases led to major harm [6].

To the best of our knowledge, no prior studies have assessed patient handoff practices among general surgery (GS) residents in Saudi Arabia. Thus, we aimed to assess the patient handoff processes among GS residents across all general surgery residency programs in Saudi Arabia. We also attempted to identify the common reasons that lead to incomplete patient handoffs resulting in patient harm as well as investigate the factors that improve patient handoffs based on the experiences of the residents.

## Methods

### Selection criteria

We conducted an anonymous survey that included all GS residents from R1 to R5 in all general surgery residency programs accredited by the Saudi Commission for Health Specialties (SCFHS). The survey was sent to the SCFHS for approval prior to its distribution among all GS residents. Individual program directors were asked to send the survey to their residents. The Research Committee of the Unit of Biomedical Ethics at King Abdulaziz University Hospital approved the study. Informed consent was obtained from all participants upon completion of the survey (Reference number 334–20). All methods were carried out in accordance with the relevant guidelines and regulations.

### Survey content

The survey was divided into four sections. The first section contained questions about demographic information, the hospital setting, and the level of residency training. The second section contained questions about verbal handoffs and how they were conducted at the residents’ institutions. The third section contained questions about written handoffs, and the fourth section contained questions about patient harm and how to improve the institutions’ existing handoff processes. A pilot study involving junior and senior GS residents at King Abdulaziz University Hospital was conducted to ensure the clarity of the survey’s content; these residents were excluded from the final analysis.

### Survey administration

The survey was administered online using SurveyMonkey (www.surveymonkey.com). The URL was sent to the GS residents through the SCFHS. To increase the response rate, the survey URL was sent directly to the surgical program directors, who then sent it to the GS residents.

#### Definition of patient handoff

Patient handoff (“sign-out”) is “the transfer of information, along with authority and responsibility, during transitions in care across the continuum, which includes an opportunity to ask questions, clarify, and confirm” [7].

#### Definition of patient harm

Minor harm was defined as an event with a limited clinical consequence owing to a communication error or incomplete handoff without causing any harm to the patient (e.g., a missed or delayed follow-up on a radiological test or laboratory result or a delay in assessing a new patient) [5]. Major harm was defined as an event with a major clinical consequence such as a complication, an injury, or even death that is secondary to a communication failure or an incomplete handoff [5].

### Statistical analysis

Data are summarized as the mean ± standard deviation for continuous variables and counts (percentages) for categorical variables. A chi-square test was conducted to test the association between the type of handoff received and the proportion of patients who suffered from minor harm.

The significance level was set at a *P* value of < 0.05 and a 95% confidence interval. Collected data were entered and analyzed with Stata version 13.0 (Stata Corp, College Station, Texas, USA).

## Results

**Fig. 1:**
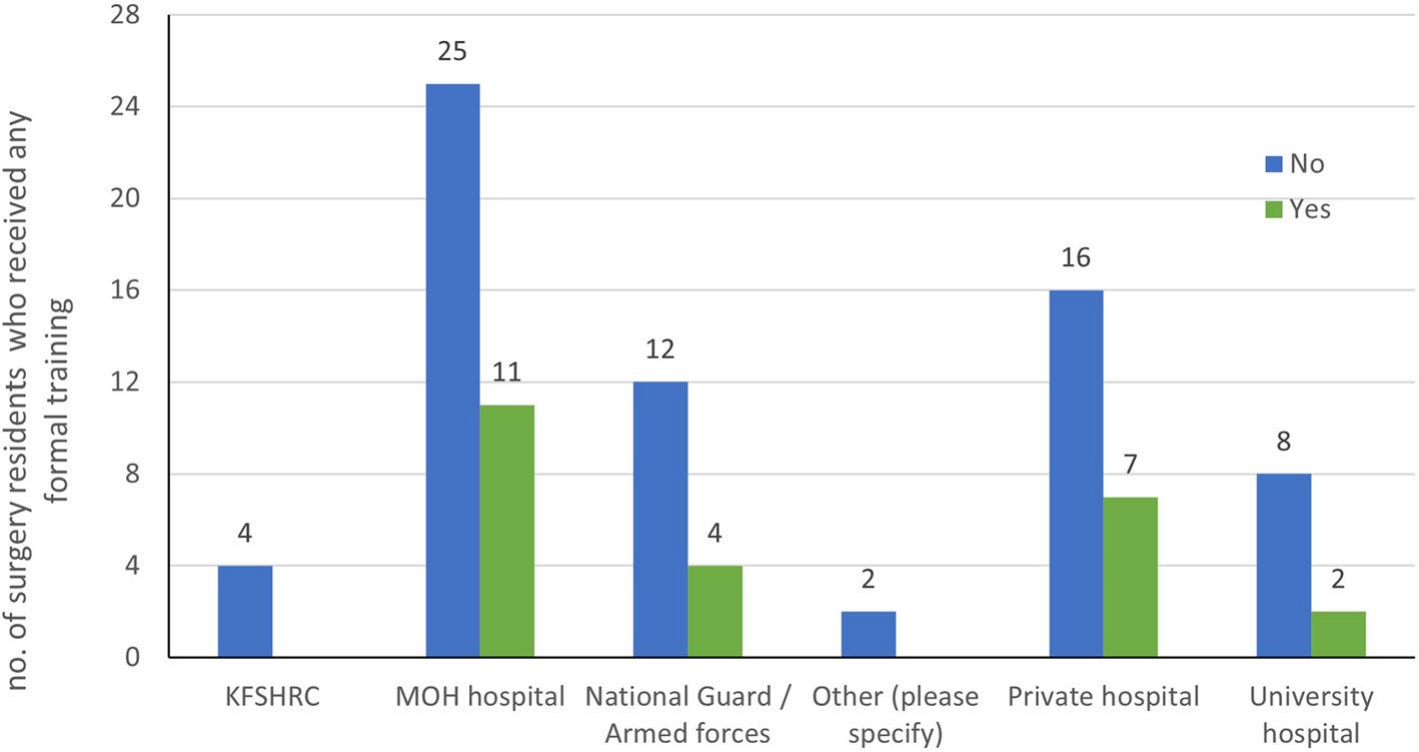
The number of surgical residents who received formal training on patient handoffs based on hospital type. KFSHRC: King Faisal Specialist Hospital and Research Center. MOH: Ministry of Health

**Fig. 2:**
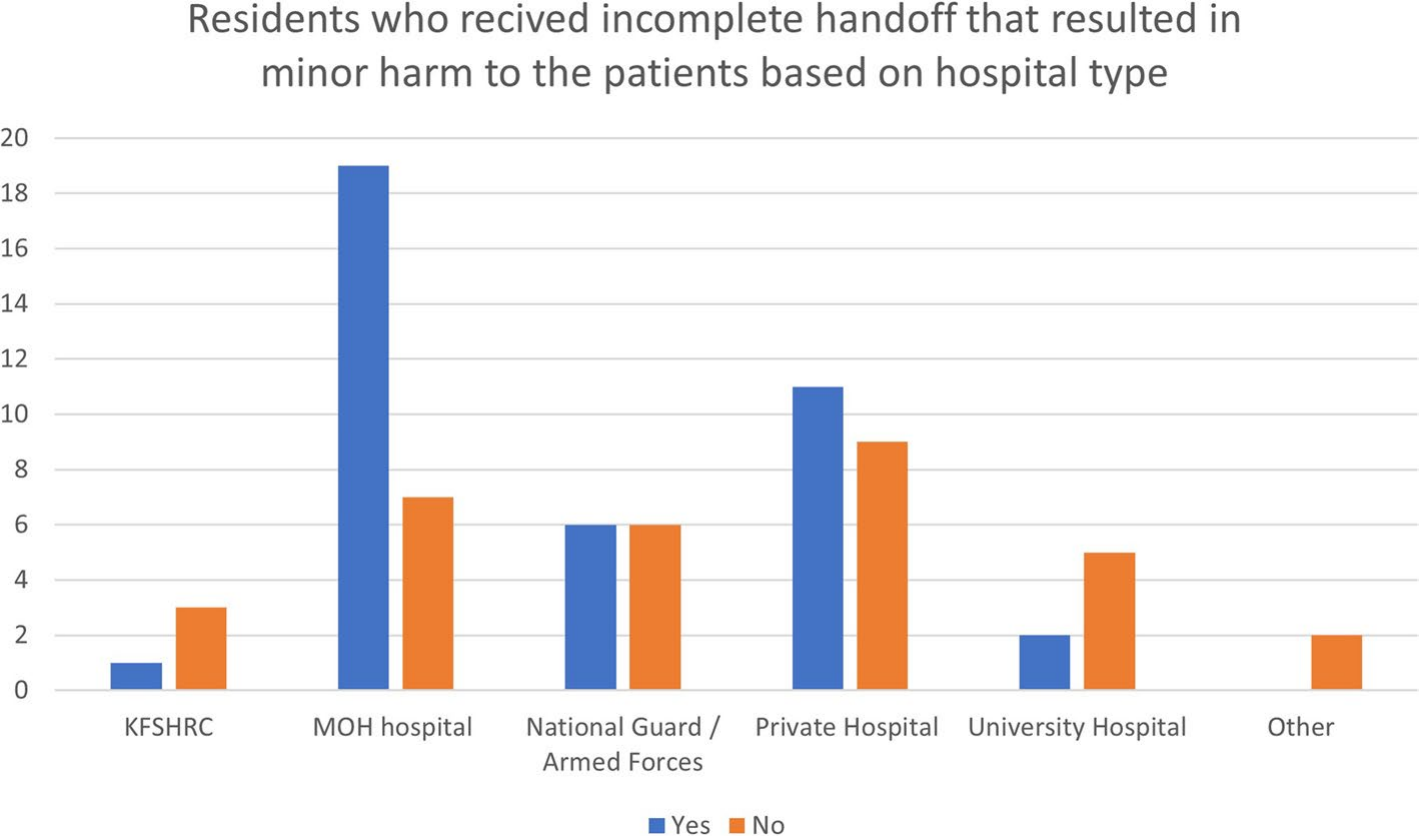
The number of residents who received incomplete patient handoff instructions that resulted in minor harm to a patient based on hospital type. KFSHRC: King Faisal Specialist Hospital and Research Center. MOH: Ministry of Health

**Fig. 3:**
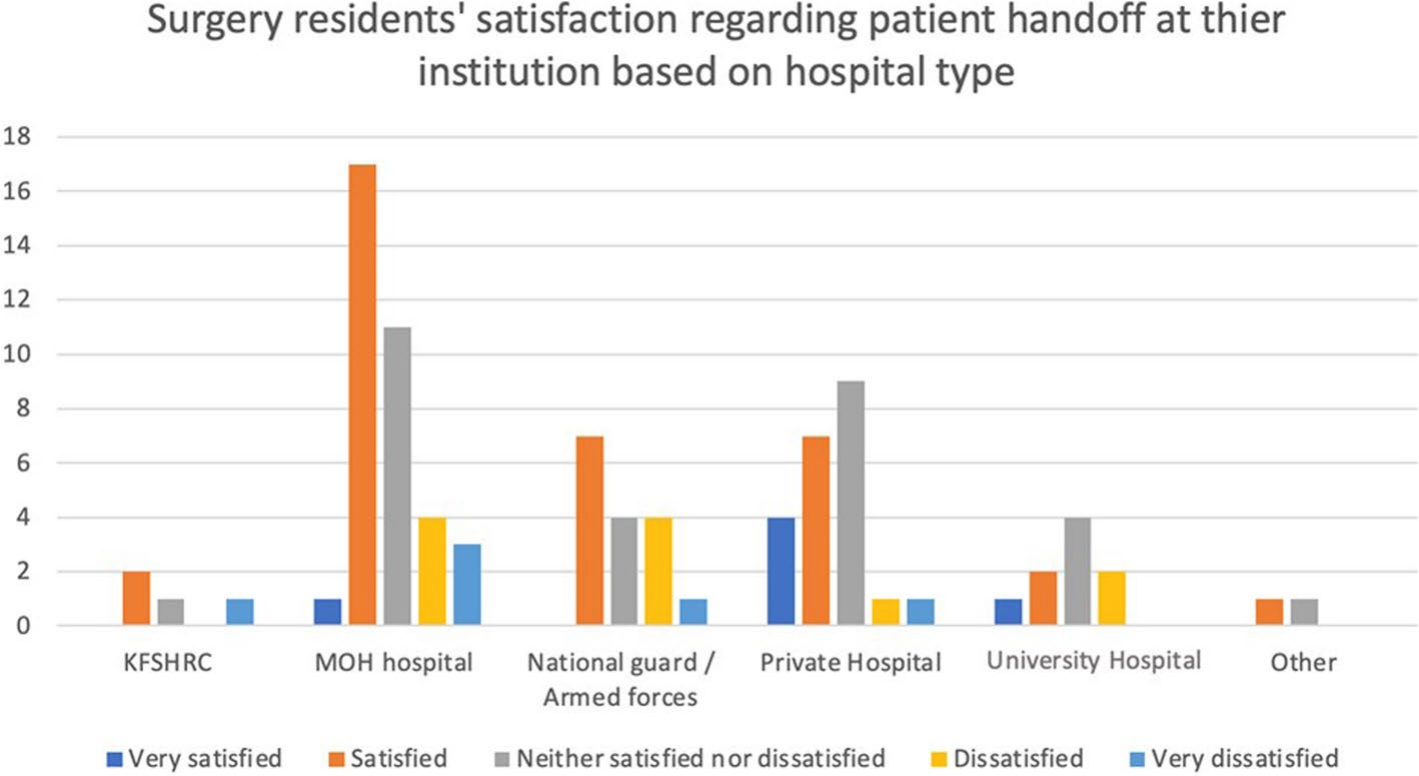
Surgical residents’ satisfaction regarding patient handoffs at their institution based on hospital type. KFSHRC: King Faisal Specialist Hospital and Research Center. MOH: Ministry of Health

A total of 118 GS residents responded, and their responses were analyzed. Table 1 presents the complete characteristics of the participants. There was an equal sex distribution, with 66 (57.3%) responses from female residents. Sixty-seven GS residents (72.8%) did not receive any formal training or attend any workshop on patient handoffs and how to conduct them. Among those who reported that they had received formal training for performing patient handoffs, 11 (45.8%) were GS residents training at the Ministry of Health hospitals. Fifty-eight (63%) GS residents believed that the time they spent during a handoff process was appropriate, while 24 (26%) believed it was too short Figs. 1, 2 and 3.

**Table 1:** Characteristics of the surgical residents who participated in the survey (*n* = 118)^a^

Characteristic	n (%)
**Sex** ^**a**^	
Male	49 (42.6%)
Female	66 (57.3%)
**Age (Median in years)**	28
**Year of internship completion**	
Before 2014	12 (10.7%)
2014	6 (5.4%)
2015	12(10.7%)
2016	16(14.2%)
2017	27(24.1%)
2018	14(12.5%)
2019	25(22.3%)
**Region**	
Northern region	0 (0%)
Southern region	0(0%)
Central region	13(11.4%)
Eastern region	4(3.51%)
Western region	97 (85%)
**Type of hospital**	
University hospital	13(11.2%)
Private hospital	26(22.4%)
Ministry of Health hospital	49(42.2%)
National guard/armed forces hospital	20(17.2%)
King Faisal Specialist Hospital and Research Center	4(3.4%)
Other	4(3.4%)
**Level of training**	
PGY1	28(24.5%)
PGY2	17(14.9%)
PGY3	19(16.6%)
PGY4	19(16.6%)
PGY5	31(27.1%)
**Residents’ level of satisfaction for patient handoffs**	
Very satisfied	6(6.6%)
Satisfied	37(41.1%)
Neither satisfied nor dissatisfied	30(33.3%)
Dissatisfied	11(12.2%)
Very dissatisfied	6(6.6%)

^a^Some of the participants skipped some of the questions, which led to the discrepancy between the total number of residents and the total number of responses

The details related to patient handoff practices among GS residents are presented in Table 2. Sixty-one (67.7%) GS residents reported that they were always or sometimes interrupted during the patient handoff process.

**Table 2:** Characteristics of patient handoffs among surgical residents in Saudi Arabia

Always	Most of the time	Sometimes	Rarely	Never	Total
**The handoff process is conducted with opportunities for questions and answers**					
29(31.5%)	23(25%)	28(30.4%)	10(10.8%)	2(2.1%)	92
**Handoffs occur at a designated time**					
26(28.2%)	32(34.7%)	21(22.8%)	9(9.7%)	4(4.3%)	92
**Handoffs occur in a designated place**					
29(31.5%)	26(28.2%)	17(18.4%)	7(7.6%)	13(14.1%)	92
**Frequency of interruptions during the handoff process**					
10(11.1%)	16(17.7%)	35(38.8%)	26(28.8%)	3(3.3%)	90

Finally, 31 (34.4%) GS residents believed that their institutions’ existing handoff systems neither adequately protected patient safety nor allowed for continuity of care. Of the GS residents who believed that the patient handoff practices at their institution were not safe, 13 (41.9%) worked at Ministry of Health hospitals, and 8 (25.8%) worked at private hospitals.

### Verbal handoffs

In total, 51 (68%) GS residents reported that they did not have a standardized protocol for a verbal handoff process at their institution, while 24 (32%) said otherwise. Tables 3 and 4 present further details on verbal patient handoffs.

**Table 3:** Characteristics of verbal patient handoff among surgery residents

Always	Most of the time	Sometimes	Rarely	Never	Total
**Do you receive verbal handoff instructions for ALL patients whom you care for while on call?**					
20(25.9%)	30(38.9%)	18(23.3%)	8(10.3%)	1(1.3%)	77
**Do you receive complete verbal handoff instructions that make you feel well-prepared for shift changes?**					
11(14.4%)	28(36.8%)	24(31.5%)	11(14.4%)	2(2.6%)	76
**Are verbal handoffs conducted face-to-face?**					
18(23.3%)	28(36.3%)	17(22%)	13(16.8%)	1(1.3%)	77
**Are verbal handoffs conducted over the phone?**					
2(2.6%)	15(19.4%)	36(46.7%)	17(22%)	7(9%)	77
**Do you use the read-back technique (repeating critical information to ensure that it has been accurately received)?**					
11(14.2%)	27(35%)	18(23.3%)	15(19.4%)	6(7.7%)	77

**Table 4:** Elements of verbal and written handoff practices reported by the surgical residents during the handoff process^a^

Characteristics	Verbal n (%)	Written n (%)
**Name of each patient**	47(61.8%)	39(72.2%)
**Age of each patient**	52(68.4%)	46 (85.1%)
**Date of admission for each patient**	27(35.5%)	39 (72.2%)
**Primary physician for each patient**	69(90.7%)	28 (51.5%)
**Type of surgical procedure(s) performed for each patient during their current admission**	66(86.8%)	50(92.5%)
**Date of procedure(s) for each patient**	48(63.1%)	39 (72.2%)
**Relevant prior surgical procedure(s) for each patient**	42(55.2%)	46(85.1%)
**Clinical course for each patient’s current admission**	48(63.1%)	33 (61.1%)
**Complications experienced by each patient**	61(80.2%)	31 (57.4%)
**Comorbidities**	55(72.3%)	31(57.4%)
**Medications for each patient**	26(34.2%)	40(74%)
**Diet information for each patient**	27(35.5%)	19(35.1%)
**Code status for each patient (if any recent change)**	29(38.1%)	18(33.3%)
**Identification of the sickest patient on the list**	47(61.8%)	22(40.7%)
**Pending laboratory results for follow-up**	59(77.6%)	25(46.3%)
**Pending consults for evaluation**	60(78.9%)	41(75.9%)
**Pending radiological tests for follow-up**	63(82.8%)	42(77.7%)
**Anticipated issues or problems**	50(65.7%)	44(81.4%)
**New consults/admissions**	47(61.8%)	33(61.1%)

^a^the frequency is different from the total participants (*n* = 75) due to missing responses for some of the characteristics

### Written handoff

GS residents reported different methods of written handoffs at their institutions. Thirty (38.9%) reported using an electronic form (such as Excel sheet or Google Sheets). Eighteen (23.3%) reported using instant messaging. Fourteen (18.1%) reported using handwritten forms, and 15 (19.4%) had no written handoff practices. Only 11 (19.3%) GS residents reported that they had received a complete written handoff about all the patients needing care during their on-call time, while 6 (10.5%) reported that they had received a complete written handoff that made them well prepared for their shift. Further details are reported in Tables 4 and 5.

**Table 5:** Characteristics of written handoff among surgery residents

Always	Most of the time	Some of the time	Rarely	Never	Total
**Do you receive complete written handoff instructions for ALL patients whom you care for while on call?**					
11(19.3%)	20 (35%)	15 (26.3%)	4 (7%)	7 (12.2%)	57
**Do you receive complete written handoff, instructions that make you feel well prepared for the shift change?**					
6 (10.5%)	22 (38.6%)	18 (31.5%)	4 (7%)	7 (12.2%)	57
**Are the written handoff instructions physically handed to you?**					
8 (14%)	11 (19.3%)	12 (21%)	10 (17.5%)	16 (28%)	57

### Patient harm

Forty (56.3%) GS residents reported that they had received incomplete handoff instructions that resulted in minor harm to a patient, and the majority were based in Ministry of Health hospitals. A total of 25 residents (64%) reported that the verbal handoff instructions did not contain the most current information, which was the most common reason for minor harm. Another reason was interruptions during patient handoffs. Furthermore, 14 (19.1%) residents were PGY-5 residents, followed by 7 (12.3%) residents who were PGY-3 residents; 26 (66.6%) discussed incomplete handoffs with the resident responsible to prevent such incidents from occurring again, while 13 (33.3%) did not discuss such events, often to avoid confrontation with their colleagues. Only 6 (8.7%) GS residents reported that they had received incomplete patient handoff instructions that resulted in major harm to a patient. Five (83.3%) GS residents who reported incidents of major harm owing to incomplete handoffs discussed the adverse events with the residents at fault. Only one (16.6%) of the GS residents did not discuss the event with colleagues to avoid confrontation. Among the incidents of major harm, three (50%) residents reported some changes in the handoff process to prevent such an incident from occurring again.

The most common reason for interruptions was medical personnel paging. Other reasons included “side conversations” with other colleagues, the late arrival of residents, which caused interruptions and required repeating the handoffs, and being needed in the operating room. The GS residents perceived that interruptions affected the handoffs: 60 (68.1%) believed that interruptions led to a decreased quality of effective communication; 39 (44.3%) believed interruptions decreased the quality of patient care; 63 (71.5%) believed that interruptions led to loss of information; and 16 (18.1%) believed interruptions led to patient harm. During on-call time, 34 (37.7%) GS residents attended to an average of 20–39 patients during their on-call period; 32 (35.5%) attended to an average of 11–19 patients; and only 8 (8.8%) covered more than 40 patients during their shift.

The GS residents also reported factors that could improve their institutions’ handoff processes, including standardizing the verbal and written handoff, and implementing an educational material about the handoff process Table 6 and Table 7.

**Table 6:** Reasons that led to an incomplete patient handoff resulting in minor and major harm.

**Reasons**	Minor harm *N* (%)	Major harm *N* (%)
The verbal handoff instructions did not contain the most current information	25(64.1%)	6(100%)
The written/electronic handoff instructions did not contain the most current information	18(46.1%)	4(66.6%)
Interruptions during the handoff process	15(38.4%)	2(33.3%)
Language barriers between the residents	2(5.1%)	1(16.6%)
Knowledge base problems from either of the residents	16(41%)	4(66.6%)
Time constraints affecting the outgoing resident	14(35.9%)	2(33.3%)
Time constraints affecting the incoming resident (you)	7(17.9%)	1 (16.6%)
Interpersonal conflicts between the incoming and outgoing residents	6(15.3%)	0(0%)
Lack of an interactive handoff process (handoff instructions given without the opportunity for questions and answers)	22(56.4%)	1(16.6%)
The handoff was conducted in a distracting environment. (e.g., hospital hallway or emergency department)	11(28.2%)	0(0%)
**Reasons why residents did not discuss incomplete handoffs resulting in minor and major harm with their colleagues**		
Avoid confrontation with their colleagues	4 (30.7%)	1 (100%)
Lack of time	3 (23%)	0
No major harm caused to the patient	2(15.3%)	0
The residents who gave the handoff instructions were more senior than the residents	2(15.3%)	0
Forgot about it	1(7.6%)	0
Overwhelmed with long call hours	1(7.6%)	0

**Table 7:** Ways to improve patient handoff based on residents’ opinions

**Residents’ suggestions to improve patient handoff**	* n* (%)
Implementing an educational course about patient handoff to all the residents	49(73.1%)
Performing handoffs under the supervision of a senior resident	40(59.7%)
Performing the handoff under supervision of an attending surgeon	31(46.2%)
Take extra measures to decrease the nonurgent interruptions during the handoff process	38(56.7%)
Standardizing written/electronic handoffs so that all residents follow the same technique to sign out every time	52(77.6%)
Standardizing verbal handoffs so all residents follow the same technique to sign out every time	43(64.1%)
Dedicating a specific time of the day for handoffs	41(61.1%)
Dedicating a specific place for handoffs to take place	31(46.2%)
Improving the electronic/written handoff computer programs	37(55.2%)
Using an electronic tablet (such as an iPad) for handoffs	25(37.3%)
Using a smartphone application for handoffs	34(50.7%)

## Discussion

Patient handoffs are essential to ensure continuity of care among health care providers. A lack of standardization in this area can make the quality of treatment variable and subjective. Multiple variables, such as patient load, the location where handoffs take place, and interruptions during handoffs, play an important role. As the number of residency programs across all specialties increases in Saudi Arabia, higher workloads and a lack of formal training and standardization are expected to increase incidents of medical errors secondary to communication failure. Effective communication is thus an essential nontechnical skill in surgeons’ manuals, where communication and teamwork are defined as “giving and receiving knowledge and information in a timely manner to aid the establishment of a shared understanding among team members” [8].

Our study showed a lack of formal training and variable quality in patient handoffs. Indeed, only 31% of the residents reported that the patient handoff was always done interactively, and less than 30% stated that it was done at the same time and in the same place. However, this result is unexpectedly better in reports from surgical residents in the U.S. and Canada [5]. The higher rates of interruptions during handoffs may also point to an environment where the patient handoff time is not regulated and protected. Health care providers can take cues from professionals in other high-risk environments where strict measures are followed to ensure clear and adequate communication. For example, in aviation, “sterile cockpit” protocols protect the cockpit crew from unnecessary distractions at different critical times during flight.

Our study shows that residents believe that major interruptions during the handoff process could have caused subsequent decreases in the quality of care. The quality of effective communication with the other health care providers was also low, and the residents believed that it often led to information loss. These results differ from those of a prospective observational study by Kitch et al. on distractions among surgery residents during patient handoffs [6], where 214 surgical resident handoffs were observed over 18 months. Although 102 (48%) residents reported distractions during patient handoffs, there was no significant difference in the quality of handoffs. This difference in quality from our results may be explained by the presence of a standardized process for both verbal and written handoffs, which made the surgical residents more resilient to distraction. This type of standardization is mostly absent in the institutions that were included in our study. However, these contrasting results may also be attributed to the Hawthorne effect; that is, the residents in Kitch et al.’s study did not provide accurate feedback on interruptions that decreased handoff quality [6]. Another retrospective study that reviewed the data of mobile phone-based paging systems among PGY1 surgical residents found that these residents received, on average, 48 ± 41 messages per shift [9]. Most of these messages were between 5.00 p.m. and 7.00 p.m., when patient handoffs and the transition of care among surgical residents take place, and no interruptions were expected, as they may lead to medical errors [10–13].

Interruptions may occur when a health care provider is trying to communicate with residents because of side conversations or due to interruptions from other residents. Fixing this problem requires standardizing a time and place for handoffs. Educating all health care providers of the “sterile hour” will reduce distractions, except in emergency cases such as during trauma codes. Criteria or a scoring system can be established to prioritize interruptions and reduce unnecessary interruptions during the sterile hour. In general, however, all unnecessary conversations should be avoided during patient handoffs and the transfer of care.

Standardization is paramount for improving the quality of patient handoffs [14, 15], and the respondents in our survey unanimously agreed with this suggestion. In our cohort, there was an obvious lack of standardization of both verbal and written handoffs, followed by incomplete verbal handoffs, which together led to the most minor and major harmful events.

To our knowledge, this is the first detailed cross-sectional study about patient handoffs among GS residents in Saudi Arabia, which is an extremely important and yet underresearched topic in this region. Finally, our study had some limitations. Our survey was restricted to only GS residents; including residents from different specialties will offer richer and more contextualized insights into the causes of and solutions for low-quality patient handoffs. In addition, the number of participants in the survey was limited.

## Conclusions

Further research is needed to assess patient handoffs across all residency programs in different specialties and in different hospital settings in Saudi Arabia to assess the true scope of the problem. Mandatory educational modules for patient handoffs should be required by the SCFHS for all residents.

## Supplementary Information

**Additional file 1.**

## Data Availability

The datasets used and/or analyzed during the current study are available from the corresponding author on reasonable request.

## Abbreviations

GS: General surgery
SCFHS: Saudi Commission for Health Specialties
KFSHRC: King Faisal Specialist Hospital and Research Center
MOH: Ministry of Health
